# Multicentre derivation and validation of a prognostic scoring system for mortality assessment in HIV‐infected patients with talaromycosis

**DOI:** 10.1111/myc.13206

**Published:** 2020-11-29

**Authors:** Yuanyuan Qin, Yihong Zhou, Yanqiu Lu, Hui Chen, Zhongsheng Jiang, Kaiyin He, Qun Tian, Yingmei Qin, Man Rao, Vijay Harypursat, Huan Li, Yaokai Chen

**Affiliations:** ^1^ Clinical Research Center Chongqing Public Health Medical Center Shapingba China; ^2^ Division of Infectious Diseases Chongqing Public Health Medical Center Shapingba China; ^3^ School of Biomedical Engineering Capital Medical University Beijing China; ^4^ Division of Infectious Diseases Liuzhou General Hospital Liuzhou Guangxi China; ^5^ Division of Infectious Diseases the Eighth People's Hospital of Guangzhou Guangzhou Guangdong China; ^6^ Division of Infectious Diseases The Third People's Hospital of Guilin Guilin Guangxi China; ^7^ Division of Infectious Diseases The Fourth People’s Hospital of Nanning Nanning China; ^8^ Division of Infectious Diseases The Third People’s Hospital of Shenzhen Shenzhen Guangdong China; ^9^ Clinical Research Center Pingdingshan Branch of Chongqing Public Health Medical Center Chongqing China

**Keywords:** AIDS, HIV, mortality, risk scoring system, Talaromycosis

## Abstract

**Background:**

Although the widespread use of modern antiretroviral therapy (ART) has reduced the incidence of talaromycosis in people living with HIV, mortality remains as high as 20% in this population, even after appropriate antifungal treatment.

**Objectives:**

The objective of our study was to develop a risk assessment system for HIV‐infected patients with comorbid talaromycosis, in order to provide these patients with appropriate, effective and potentially life‐saving interventions at an early stage of their illness.

**Patients/Methods:**

This was a multicentre, retrospective cohort study conducted in China. We built a predictive model based on data from 11 hospitals, and a validated model using the data of 1 hospital located in an endemic area.

**Results:**

Forward stepwise multivariate statistical calculations indicated that age, aspartate aminotransferase/alanine transaminase ratio and albumin levels, and BUN levels were valid, independent predictors of the risk of death in HIV‐infected patients with talaromycosis. Our developed and validated risk scoring system is effective for the identification of HIV‐infected patients with talaromycosis at high risk of death at hospital admission (*p* < .001; AUC = 0.860). In our study, our risk prediction model provided functional and robust discrimination in the validation cohort (*p* < .001; AUC = 0.793).

**Conclusion:**

The prognostic scoring system for mortality assessment developed in the present study is an easy‐to‐use clinical tool designed to accurately assist clinicians in identifying high‐risk patients with talaromycosis.

## 1 INTRODUCTION

*Talaromyces marneffei* (*T. marneffei*), formerly known as *Penicillium marneffei* (*P. marneffei*), is a dimorphic fungus that causes life‐threatening infection in immunocompromised patients, especially in endemic areas such as South‐East Asia, Southern China and India.^1^ As one of the AIDS‐defining diseases, talaromycosis represents a significant burden on patients with AIDS, and mortality and morbidity rates are second only to the mortality and morbidity wreaked by tuberculosis and cryptococcosis in Thailand.^2^ Although the widespread use of modern antiretroviral therapy has reduced the incidence of talaromycosis in people living with HIV, its mortality remains up to 20%, even after appropriate antifungal treatment.^3^, ^4^, ^5^, ^6^

Several past studies have assessed the risk factors associated with death in patients with talaromycosis.^4^, ^7^ However, there remains no clinical risk scoring tool for people living with HIV who suffer from talaromycosis. Providing a risk scoring system to identify high‐risk patients with talaromycosis is useful in the clinical care of patients with AIDS. The objective of our study was to establish a clinical risk assessment system for patients with talaromycosis in order to provide appropriate, effective and life‐saving interventions in this population at an early stage of their illness.

## 2 MATERIALS AND METHODS

### 2.1 Study design and patient selection

This study was a multicentre, retrospective cohort study conducted in China. HIV‐infected patients with a diagnosis of talaromycosis were enrolled in this study from the following 11 hospitals from around China: Chongqing Public Health Medical Center, Beijing Youan Hospital, Harbin Medical University, the Second People's Hospital of Tianjin, Liuzhou General Hospital, the Third People's Hospital of Guilin, the Third People's Hospital of Shenzhen, Yunnan Provincial Infectious Disease Hospital, the Fourth People's Hospital of Nanning, Guangxi Longtan Hospital and Xixi Hospital of Hangzhou. The study was approved by The Ethics Committee of the Chongqing Public Health Medical Center (2019‐003‐02‐KY). The requirement of informed consent from study participants was waived by the ethics committees because of the retrospective and anonymized nature of this study. Data from these patients were used to populate the derivative cohort. The validation cohort comprised data from HIV‐infected patients with a diagnosis of talaromycosis enrolled at the Eighth People's Hospital of Guangzhou.

#### 2.1.1 The derivative cohort

We analysed the clinical and demographic data of 384 cases of talaromycosis from 11 Chinese hospitals between 2010 and 2019. Demographics, clinical manifestations and laboratory test results at admission were collected and analysed for the development of a risk prediction model of the risk of mortality in HIV‐infected patients with talaromycosis.

#### 2.1.2 The validation cohort

To verify the applicability for broad implementation, and validity of our derived prediction model, we further analysed the demographics, clinical manifestations and laboratory test results at admission of 233 cases of confirmed talaromycosis admitted to the Eighth People's Hospital of Guangzhou between 2015 and 2016.

### 2.2 Data sources

Demographic and clinical characteristics of patients with talaromycosis in the derivative and validation cohorts were transcribed from electronic hospital medical record systems onto the Medical Research Platform (http://www.51yyt.org/FrontPage/login.aspx?Inviter=). The data for the validation cohort were extracted from the data of patients with talaromycosis admitted to the Eighth People's Hospital of Guangzhou. Patients lacking the necessary data in Medical Research Platform were excluded from the final analysis.

### 2.3 Definitions

A confirmed diagnosed case of talaromycosis met one of following criteria: (a) *T. marneffei* found in histological biopsy specimens, and (b) *T. marneffei* cultured from clinical specimens.

### 2.4 Predictor variables

We explored demographic factors, comorbid illnesses, symptoms, signs and laboratory test results at admission, including haemoglobin levels, platelet counts, white blood cell counts, aspartate aminotransferase/alanine transaminase (AST/ALT) ratios, albumin levels, total bilirubin levels, creatinine levels, lactate dehydrogenase (LDH), CD4^+^ T‐cell counts and blood urea nitrogen (BUN) levels, in order to select surrogate variables for risk of death in patients with talaromycosis.

### 2.5 Statistical analysis

In the derivative cohort, the demographic, clinical and laboratory variables of patients were analysed at the time of admission by standard descriptive statistics. Continuous variables were described using median with interquartile ranges (IQR). Categorical variables were described as frequency rates and percentages. Univariate analysis was performed using logistic regression to explore factors associated with death. Predictors with continuous variables in the univariate analysis (*p* ≤ .1) were converted to categorical variables by grouping values using cut‐off points based on either the median, or clinically relevant values, and each point score was assigned for each categorical variable. To achieve the scoring sums, age, AST/ALT ratio, albumin level were first included separately in a logistic regression model. Factors that were significant in the step were simultaneously placed into a single logistic regression model. We used the variance inflation factor (VIF) to make assessments of multicollinearity among the independent variables, but there were not any. From the logistic regression models, the co‐efficient scores were assigned for each factor with the respective β coefficients. The co‐efficient scores in the results were rounded off to the nearest 0.5. A specific risk score is derived by a calculation of each co‐efficient score multiplied by each point score. The score for an individual was obtained by summing the scores for the risk score of each of the risk factors.

To verify the validity of the scoring system, we plotted receiver operating characteristic (ROC) curves for patients in the derivative cohort and those in the validation cohort respectively, and compared the areas under these ROC curves.

All analyses were performed using Statistical Package for the Social Sciences software, Version 18.0 (IBM‐SPSS Statistics, Chicago, IL, USA) and MedCalc, Version 12.7.1.0 (MedCalc software, Mariakerke, Belgium).

## 3 RESULTS

**Figure 1:**
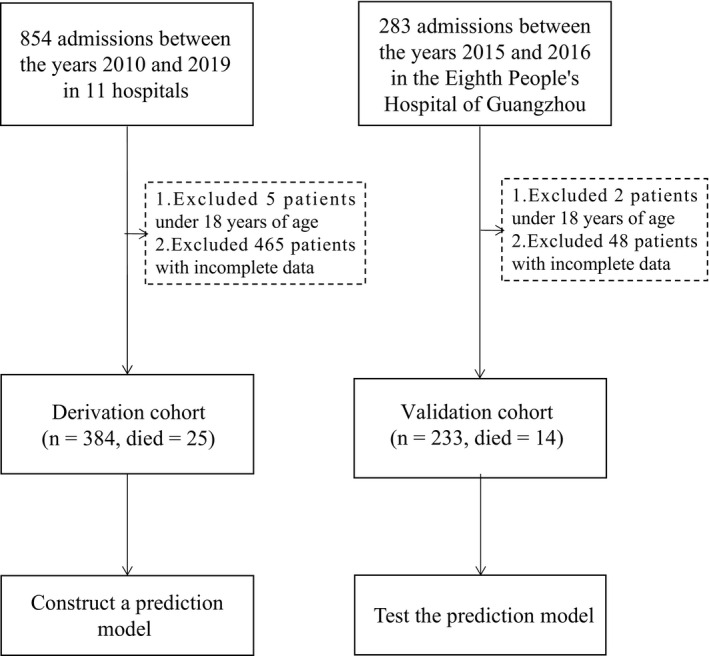
Flow chart of the study population

A total of 1137 cases of HIV‐positive patients with talaromycosis were screened in this study from the 12 included hospitals. Seven patients were excluded for being under 18 years of age, and 513 patients were excluded for data loss. Figure 1 provides a flowchart for patient inclusion and available data for analysis.

Of the patients in the derivative cohort, 25 died during hospitalization, while in the validation cohort, 14 patients succumbed to talaromycosis during hospitalization, respectively. Table 1 presents the demographic characteristics, symptoms and signs, comorbidities, and laboratory results of HIV‐infected patients with talaromycosis in the derivative cohort.

**Table 1:** Univariate analysis of baseline cohort characteristics of HIV‐infected patients with talaromycosis in the derivative cohort

Characteristics	Total (n = 384)	Survivor (n = 291)	Non‐Survivor (n = 25)	*p*	Univariate effect: OR
Socio‐demographic					
Medical age, median (IQR), years	47.5 (38‐57)	47 (38‐56)	57 (36.5‐70.5)	.016	1.037 (1.007‐1.068)
Female sex, n (%)	76 (19.8)	68 (18.9)	8 (32.0)	.119	2.014 (0.835‐4.859)
Injection drug user, n (%)	8 (2.1)	7 (1.9)	1 (4.0)	.497	2.095 (0.248‐17.732)
ART at hospital admission, n (%)	45 (11.7)	44 (12.3)	1 (4.0)	.242	3.352 (0.442‐25.400)
Time from symptom onset to hospital admission, median (IQR), month	1 (0.4‐1.5)	1 (0.4‐1.4)	1 (0.4‐2.0)	.680	0.951 (0.747‐1.209)
Pneumocystis pneumonia, n (%)	53 (13.8)	46 (12.8)	7 (28.0)	.039	2.646 (1.048‐6.682)
Herpes simplex infection, n (%)	10 (2.6)	8 (2.2)	2 (8.0)	.102	3.815 (0.766‐19.008)
Tuberculosis, n (%)	38 (9.9)	36 (10.0)	2 (8.0)	.743	0.780 (0.177‐3.446)
Bacterial pneumonia, n (%)	86 (22.4)	78 (21.7)	8 (32.0)	.238	1.695 (0.705‐4.075)
Hepatitis B, n (%)	42 (10.9)	40 (11.1)	2 (8.0)	.628	0.693 (0.158‐3.052)
Symptoms and signs					
Fever, n (%)	296 (77.1)	277 (77.2)	19 (76.0)	.894	0.937 (0.362‐2.425)
Dyspnoea, n (%)	78 (20.3)	72 (20.1)	6 (24.0)	.636	1.259 (0.485‐3.266)
Skin lesions, n (%)	105 (27.3)	97 (27.0)	8 (32.0)	.590	1.271 (0.531‐3.040)
Weight loss	267 (69.5)	250 (69.6)	17 (68.0)	.863	0.927 (0.388‐2.211)
Median pulse (beats/minute, IQR)	99.0 (87.0‐114.0)	98.0 (86.0‐113.0)	106.0 (92.0‐121.0)	.089	1.018 (0.997‐1.038)
Mean arterial pressure (mm Hg, IQR)	81.3 (73.3‐90.0)	81.7 (73.7‐90.0)	76.7 (69.0‐91.0)	.074	0.967 (0.933‐1.003)
Laboratory results					
Mean haemoglobin (g/L, IQR)	93.0 (79.0‐109.0)	94.0 (79.0‐110.0)	78.0 (61.5‐87.0)	<.001	0.964 (0.944‐0.983)
Median platelets (×10^9^/L, IQR)	120.0 (54.3‐193.8)	121.0 (59.0‐196.0)	77.0 (29.5‐129.0)	.008	0.992 (0.986‐0.998)
Median white blood cell count (×10^9^/L, IQR)	3.7 (2.6‐5.4)	3.7 (2.5‐5.4)	4.1 (2.8‐5.7)	.853	0.999 (0.994‐1.005)
Median AST/ALT(IQR)	2.1 (1.5‐3.5)	2.0 (1.4‐3.3)	4.0 (3.0‐6.2)	<.001	1.302 (1.143‐1.483)
Median albumin (g/L, IQR)	26.2 (22.3‐30.4)	26.5 (23.0‐30.8)	20.4 (16.8‐24.6)	<.001	0.802 (0.732‐0.878)
Median total bilirubin (μmol/L, IQR)	9.7 (7.2‐15.5)	9.5 (7.1‐14.9)	13.0 (9.3‐20.1)	.363	1.010 (0.988‐1.033)
Median creatinine (μmol/L, IQR)	72.2 (59.0‐89.0)	71.8 (59.0‐87.8)	77.0 (61.6‐108.5)	.033	1.006 (1.000‐1.011)
LDH (U/L, IQR)	358.4 (264.8‐552.7)	354.0 (258.0‐549.0)	472.0 (328.2‐857.9)	.005	1.001 (1.000‐1.001)
CD4 + T‐cell counts (cells/μL, IQR)	14 (7‐27)	14.0 (7.0‐27.0)	16.0 (7.5‐26.5)	.472	0.995 (0.981‐1.009)
BUN (mmol/L, IQR)	4.4 (3.3‐6.1)	4.3 (3.3‐5.9)	6.9 (5.0‐11.2)	<.001	1.149 (1.074‐1.228)

Abbreviations: ALT, Alanine transaminase; AST, Aspartate aminotransferase; BUN, Blood urea nitrogen; IQR, Interquartile range; LDH, Lactate dehydrogenase.

### 3.1 Construction of the Predictive Model using the derivation cohort

We assigned ‘yes’ a value of 1, and ‘no’ a value of 0, as point scores to the dichotomous variables in Table 1. All continuous variables in Table 1 associated with mortality in the univariate analysis (*p* ≤ .1) were converted into dummy variables. The assignment of these values is shown in Table 2.

**Table 2:** Point scores of 384 HIV‐infected patients with talaromycosis in the derivation cohort

	0	1	2	3	4
Age	<45	45–59.9	60–74.9	≥75	
Time from symptom onset to hospital admission in months	<1	1–2	>2		
Pulse, beats/min	<60	60–100	101–130	>130	
Mean arterial pressure levels, mm Hg	<70	70–105	>105		
Haemoglobin levels, g/L	≥90	89.9–60	<60		
Platelet levels, ×10^9^/L	≥150	100–149.9	50–99.9	20–49.9	<20
White blood cell counts, ×10^9^/L	<4	4–11	>11		
AST/ALT ratio	≤1	1.1–2	>2		
Albumin levels, g/L	≥35	25–34.9	18–24.9	<18	
Total bilirubin levels, μmol/L	<20	20–32.9	33–101.9	≥102	
Creatinine levels, mg/L	<110	110–170	170.1–299.9	300–440	>440
LDH levels, U/L	<250	250–350	>350		
BUN levels, mmol/L	<3	3–7	7.1–17	>17	

Abbreviations: ALT, Alanine transaminase;AST, Aspartate aminotransferase; BUN, Blood urea nitrogen; LDH, Lactate dehydrogenase.

All dummy variables associated with mortality in the univariate analysis (*p* ≤ .1), were included in a logistic regression model. Forward stepwise multivariate analysis indicated that age, AST/ALT ratios, and albumin levels were independent predictors of the risk of death in HIV‐infected patients with talaromycosis. The co‐efficient scores in the results were rounded off to the nearest 0.5. The details of all significant surrogates in the final stepwise logistic regression model are listed in Table 3.

**Table 3:** Independent predictors in HIV‐infected patients with talaromycosis in the derivative cohort

	Co‐efficient	Score of co‐efficient	*p*	Multiple logistic regression: OR	95% CI
Age	0.673	1	.005	1.960	1.225–3.136
AST/ALT ratio	2.535	3	.015	12.620	1.653–96.360
Albumin levels	1.189	1.5	.001	3.285	1.605–6.723

Abbreviations: ALT, Alanine transaminase; AST, Aspartate aminotransferase.

A specific risk score is derived by a calculation of each co‐efficient score (Table 3) multiplied by each point score (Table 2), applied as follows: (Age × 1) + (AST/ALT ratio × 3) + (Albumin level × 1.5). Table 4 shows the risk surrogates in the final logistic regression model, alongside their associated score component values.

**Table 4:** Hospital admission risk surrogates and associated score component values

Risk markers	Score component value
Age	
<45	0
45–59.9	1
60–74.9	2
≥75	3
AST/ALT ratio	
≤1	0
1.1–2	3
>2	6
Albumin level	
≥35	0
25–34.9	1.5
18–24.9	3
<18	4.5

Abbreviations: ALT, Alanine transaminase;AST, Aspartate aminotransferase.

### 3.2 Model assessment in the derivative cohort

**Figure 2:**
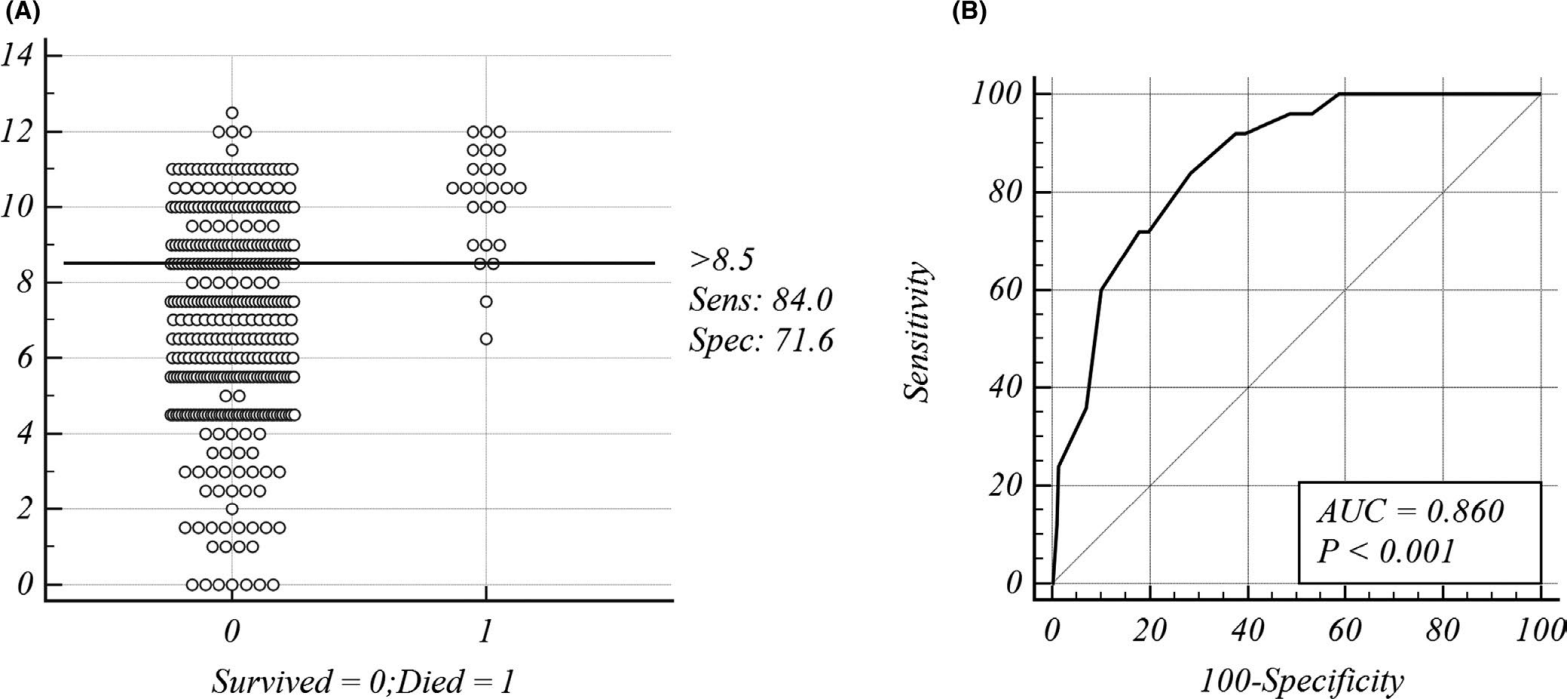
Mortality risk prediction rules for 384 HIV‐associated talaromycosis cases in the derivation cohort. A, Interactive dot diagram. The total score calculated by the prediction model labels the vertical axis of the above plot, and the clinical outcome of a patient during hospitalization marks the horizontal axis. The score of each patient were displayed as dots on two vertical axes. The horizontal line indicates the cut‐off point with the best separation. When using a score of 8.5 as the cut‐off value to predict the mortality risk of patients with talaromycosis, the sensitivity of our scoring system was 84.0% (95% CI, 0.639‐0.955), its specificity was 71.6% (95% CI, 0.666‐0.762). B, Receiver operator characteristic (ROC) curves. Each point on the ROC curve represents a sensitivity/specificity pair corresponding to a particular decision threshold. The area under the ROC curve is a measure of how well a parameter can distinguish between two groups (survived/died). AUC, area under the ROC curve; Sens, sensitivity; Spec, specificity

Our scoring system is valid for the identification of HIV‐infected patients with talaromycosis at high risk of death on hospital admission (*p* < .001; AUC = 0.860; Figure 2). We chose a total score greater than 8.5 as the predictive value for high‐risk patients. When using a score of 8.5 as the cut‐off value to predict the mortality risk of patients with talaromycosis, the sensitivity of our scoring system was 84.0% (95% CI, 0.639–0.955), its specificity was 71.6% (95% CI, 0.666–0.762), its positive predictive value (PPV) was 17.1% (95% CI, 0.140–0.207), its negative predictive value (NPV) was 98.5% (95% CI, 0.96.3–0.994), and its Youden index, which measures the performance of our scoring system, was 55.59% (Additional file 1: Table S1).

To facilitate the application of this predictive model in the clinic, we categorized the patients with these risk factors into the following 2 groups: a ‘low’‐risk group (≤8.5 score) and a ‘high’‐risk group (>8.5 score). The mortality rate increased rapidly as the scores increased. In the derivation cohort, the mortality rate was significantly higher in the ‘high’‐risk group (21/123, 20.2%) than that in the ‘low’‐risk group (4/261, 1.1%) (*p* < .001), as shown in Table 5.

**Table 5:** Predicted probability of mortality of HIV‐infected patients with talaromycosis in the derivation cohort and validation cohort

Degrees	Score	Survived	Died	*p*‐value
Derivation cohort				
Low risk	≤8.5	257 (98.9%)	4 (1.1%)	<.001
High risk	>8.5	102 (79.8%)	21 (20.2%)	
Validation cohort				
Low risk	≤8.5	159 (98.1%)	3 (1.9%)	<.001
High risk	>8.5	60 (84.5%)	11 (15.5%)	

### 3.3 Model assessment in the validation cohorts

**Figure 3:**
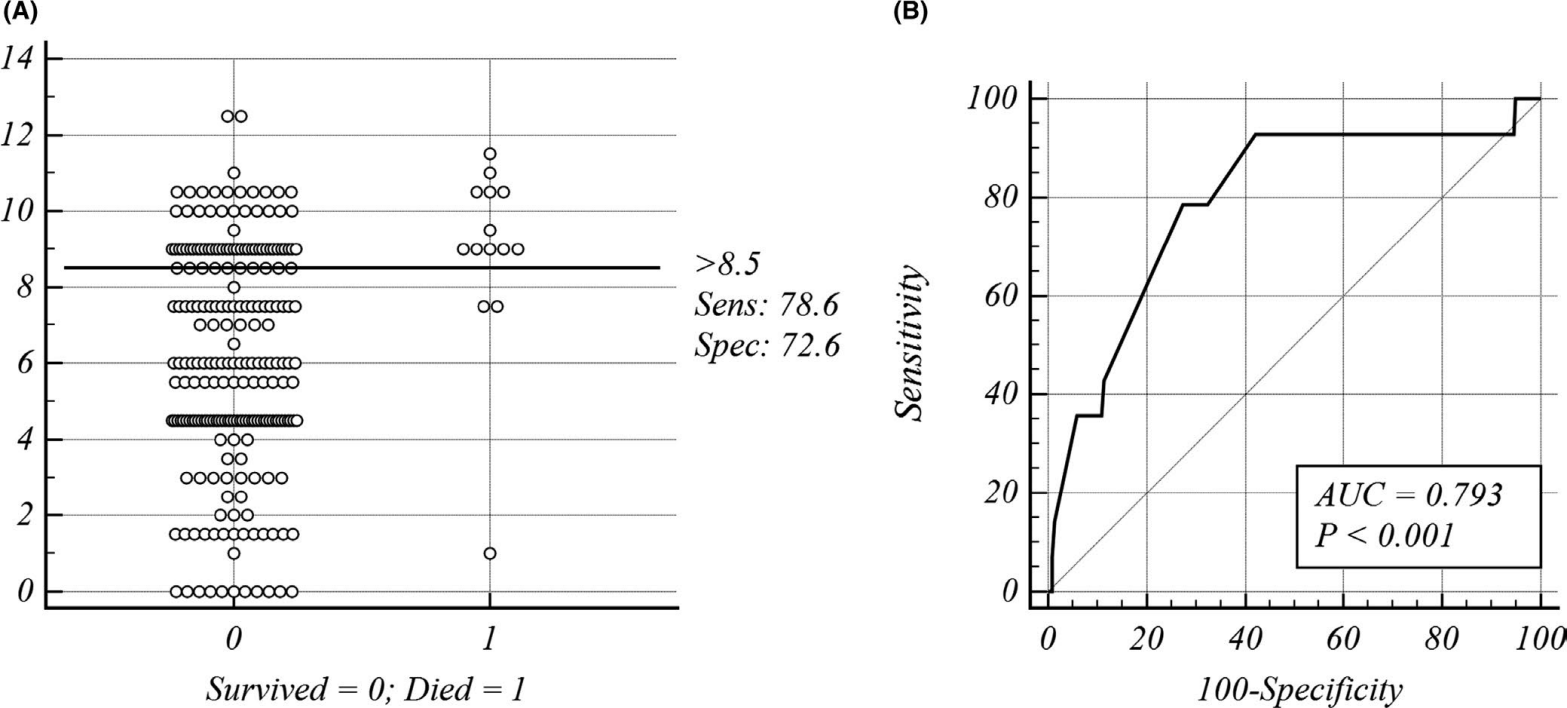
Mortality risk prediction rules for 233 HIV‐associated talaromycosis cases in validation cohort. A, Interactive dot diagram. The total score calculated by the prediction model labels the vertical axis of the above plot, and the clinical outcome of a patient during hospitalization marks the horizontal axis. The score of each patient were displayed as dots on two vertical axes. The horizontal line indicates the cut‐off point with the best separation. When using a score of 8.5 as the cut‐off value to predict the mortality risk of patients with talaromycosis, the sensitivity of the risk scoring system was calculated to be 78.6% (95% CI, 0.492‐0.953), its specificity was 72.6% (95% CI, 0.662‐0.784). B, Receiver operator characteristic (ROC) curves. Each point on the ROC curve represents a sensitivity/specificity pair corresponding to a particular decision threshold. The area under the ROC curve is a measure of how well a parameter can distinguish between two groups (survived/died). AUC, area under the ROC curve; Sens, sensitivity; Spec, specificity

In the validation cohort, the prediction model provided robust discrimination (*p* < .001; AUC = 0.0.793; Figure 3). Sensitivity of the risk scoring system was calculated to be 78.6% (95% CI, 0.492–0.953), its specificity was 72.6% (95% CI, 0.662–0.784), its PPV was 15.5% (95% CI, 0.115–0.206), its NPV was 98.1% (95% CI, 0.951–0.993), and the Youden index was 51.17%. In the validation cohort, the mortality rate was significantly higher in the high‐risk group (11/71, 15.5%) than that in the low‐risk group (3/162, 1.9%) (*p* < .001), as shown in Table 5.

## 4 DISCUSSION

The present multicentre, retrospective study included cohorts from endemic and non‐endemic areas of China. In the present study, the overall in‐hospital mortality in the three cohorts was 6.4%, which is relatively lower than that found in other studies,^3^, ^5^, ^8^ and which include mortality rates for patients discharged from hospital with longer follow‐up times.

As expected, elderly patients with HIV showed a higher risk of succumbing to talaromycosis during hospitalization. Unlike other cohorts in studies similar to ours,^3^, ^7^, ^9^ the patients in the present study were relatively older. This allowed older age to be assessed as one of the important variables for predicting prognosis in talaromycosis patients. Currently, people living with HIV survive to a much older age globally, and thus, in this population, the predictive value of increasing age on prognosis should not be ignored.

When a patient presents with typical talaromycosis skin lesions, clinicians may make a suspected diagnosis of talaromycosis. Conversely when a patient presents with systemic infection without skin lesions, a diagnosis of talaromycosis is not usually considered. Kuntipong et al reported six hepatic talaromycosis cases without skin lesions in Thailand.^10^ Regrettably, only 2 of 6 patients received amphotericin B treatment in a timely fashion, and subsequently recovered. A study by Thuy Le et al found that hepatic transaminases are often elevated in patients with talaromycosis, and that the ratio of AST to ALT in patients with talaromycosis is approximately 2.^3^ However, their study did not investigate whether elevated liver transaminases are specifically associated with mortality in patients with talaromycosis. Another study from Vietnam found that fatal talaromycosis cases have a higher average AST level than nonfatal cases.^7^ We included AST/ALT in our forward stepwise multivariate logistic regression analysis, and the results indicate that as the ratio increases, the risk of death for patients with talaromycosis also increased. Hypoproteinemia is often a manifestation of a patient's progression to cachexia. Our study found that lower albumin levels are an indicator of poor prognosis.

Many risk factors for death have been reported in patients with talaromycosis. A retrospective study conducted in Shanghai (unfortunately with a relatively small sample size) indicated that low C0D4^+^ T‐cell counts and low haemoglobin levels were associated with higher mortality of talaromycosis among HIV‐infected patients.^11^ A recent study indicated that CD4^+^ T‐cell counts and CD4+/CD8^+^ ratios are critical determinants of prognosis in *T. marneffei*‐infected patients with respiratory system lesions.^12^ However, most patients in these cohorts had CD4^+^ T‐cell counts less than 50 cells/μL, and there was no statistically significant difference in CD4^+^ T‐cell counts between the survival group and the non‐survival group. Even though some patients had initiated ART before hospitalization, their mortality rates were calculated to be not statistically distinct from ART‐naive patients. Thus, we speculated that the majority of talaromycosis cases occur in ART‐naive patients with low immune function, and the vitality of immune function of these patients does not seem to contribute to mortality during hospitalization in a clinically appreciable manner.

The risk assessment scale designed in this study can distinguish high‐risk patients from low‐risk patients to a certain extent, especially for low‐risk patients. Since the definitive diagnosis of talaromycosis mainly relies on the identification of the aetiologic organism by culture, and the return of the culture results usually takes a few days, physicians often need to decide on whether to initiate empirical antifungal treatment on not. Initiating empirical antifungal treatment for mortality low‐risk patients would increase their pharmaceutical and economic burden. With the help of our scoring assessment model, it will be easier for clinicians to a more appropriate decision.

We included all potential risk factors with *p*‐values < .1 in the construction of our risk prediction model. Our statistical analysis indicates that age, AST/ALT ratio, and albumin levels are significantly associated with mortality in patients with talaromycosis. Creation of a clinical scoring system by developing a prognostic model incorporating these variables is likely to be of great practical value in attempting to accurately identify patients at differing risks of mortality from talaromycosis during hospitalization. Whether low‐risk patients need to start antifungal treatment immediately before definitive diagnosis is an important question that warrants further study. If such patients could start antifungal therapy after definitive diagnosis, it may reduce the occurrence of untoward adverse events caused by initiation of empirical antifungal therapy. Moreover, assessment of the risk for mortality in patients with talaromycosis may provide a timely warning to attending physicians, encouraging closer monitoring of higher‐risk patients, and expeditious access to advanced rescue measures.

The results of analysis of our data in the validation cohort indicated that our risk scoring model is robust and efficacious in distinguishing HIV‐infected patients with talaromycosis at different risks of mortality. From our data, we have ample reason to believe that patients with a risk score greater than 8.5 have a significantly higher risk of death, and clinicians should therefore pay reasonable and adequate heed to this subset of patients in their quest to diminish mortality. Conversely, patients with a score of less than 8.5 have a relatively lower risk of death, which can support the call to only initiate anti‐talaromycosis treatment after definitive diagnosis is confirmed in order to avoid unnecessary drug use. Specifically, in patients with suspected renal dysfunction, our novel risk assessment model may be useful in potentially ameliorating the possible nephrotoxicity caused by amphotericin B on the one hand, and the life‐threatening infection caused by *T. marneffei* on the other.

Our study cohort enrolled a large number of patients with talaromycosis from multiple centres throughout China. Nevertheless, there are limitations to our study. Firstly, although we used geographical and temporal divisions as criteria to separate the derivation from the validation sets, validation based on a prospective cohort is generally recommended for this type of study. Secondly, we conducted our risk prediction analysis based only on in‐hospital mortality, and did not continue to follow‐up patients after hospital discharge. The Itraconazole versus Amphotericin B for Penicilliosis trial emphasized that it is important to conduct prolonged follow‐up for patients with talaromycosis participating in antifungal therapy trials in order to accurately estimate actual mortality rates.^13^ Therefore, the investigation of potential tools to accurately calculate longer‐term mortality risk may be warranted. Lastly, this study was conducted using data from patients of Chinese nationality exclusively. It is unknown whether the results of our study may safely be extrapolated to population groups in other endemic areas. Further studies in this regard in other‐than‐Chinese populations are warranted.

In conclusion, the prognostic scoring system for mortality assessment developed in the present study is an easy‐to‐use tool designed to assist attending clinicians in the rapid and accurate stratification between low‐risk and high‐risk patients with talaromycosis, thereby assisting clinicians to formulate an expeditious and effective treatment strategy based on the patients’ risk of mortality. The novel clinical risk scoring system is likely to facilitate closer clinical monitoring of talaromycosis patients at higher risk of mortality, and to provide support for empirical antifungal treatment in high‐risk patients, and also to support clinical decisions to prescribe precise antifungal treatment in low‐risk patients with a definitive diagnosis.

## CONFLICT OF INTEREST

All authors declare that they have no competing interests.

## AUTHOR CONTRIBUTION

**Yuanyuan Qin:** Conceptualization (equal); Data curation (equal); Formal analysis (equal); Writing‐original draft (equal). **Yihong Zhou:** Conceptualization (equal); Methodology (equal); Visualization (equal); Writing‐original draft (equal). **Yanqiu Lu:** Data curation (equal); Methodology (equal); Software (equal). **Hui Chen:** Methodology (equal); Software (equal); Writing‐review & editing (equal). **Zhongsheng Jiang:** Project administration (equal); Resources (equal). **Kaiyin He:** Project administration (equal); Resources (equal). **Qun Tian:** Project administration (equal); Resources (equal). **Yingmei Qin:** Project administration (equal); Resources (equal). **Man Rao:** Investigation (equal); Resources (equal). **Vijay Harypursat:** Writing‐review & editing (equal). **Huan Li:** Supervision (equal); Writing‐review & editing (equal). **Yaokai Chen:** Project administration (equal); Supervision (equal); Writing‐review & editing (equal).

## Supporting information

Table S1

## Data Availability

The data that support the findings of this study are available from the corresponding author upon reasonable request
