# Supplementary material for: Multicentre derivation and validation of a prognostic scoring system for mortality assessment in HIV‐infected patients with talaromycosis
Source: Mycoses. 2020 Nov 29;64(2):203–11. doi: 10.1111/myc.13206 (PMC7839706; doi:10.1111/myc.13206)
Supplement: Supplementary file 1 — Table S1 [file MYC-64-203-s001.docx]

Table S1 Test characteristics of mortality prediction rules for 384 HIV-associated patients with talaromycosis

| Criterion | Sensitivity | 95% CI | Specificity | 95% CI | Positive Likelihood Ratio | Negative Likelihood Ratio | Positive predictive value  (%) | 95% CI | Negative predictive value  (%) | 95% CI |
| --- | --- | --- | --- | --- | --- | --- | --- | --- | --- | --- |
| ≥0 | 100.00 | 86.3 - 100.0 | 0.00 | 0.0 - 1.0 | 1.00 |  | 6.5 | 6.5 - 6.5 |  |  |
| >0 | 100.00 | 86.3 - 100.0 | 1.95 | 0.8 - 4.0 | 1.02 | 0.00 | 6.6 | 6.5 - 6.7 | 100.0 |  |
| >1 | 100.00 | 86.3 - 100.0 | 3.06 | 1.5 - 5.4 | 1.03 | 0.00 | 6.7 | 6.6 - 6.8 | 100.0 |  |
| >1.5 | 100.00 | 86.3 - 100.0 | 5.29 | 3.2 - 8.1 | 1.06 | 0.00 | 6.8 | 6.7 - 7.0 | 100.0 |  |
| >2 | 100.00 | 86.3 - 100.0 | 5.57 | 3.4 - 8.5 | 1.06 | 0.00 | 6.9 | 6.7 - 7.0 | 100.0 |  |
| >2.5 | 100.00 | 86.3 - 100.0 | 6.96 | 4.6 - 10.1 | 1.07 | 0.00 | 7.0 | 6.8 - 7.1 | 100.0 |  |
| >3 | 100.00 | 86.3 - 100.0 | 9.19 | 6.4 - 12.7 | 1.10 | 0.00 | 7.1 | 6.9 - 7.3 | 100.0 |  |
| >3.5 | 100.00 | 86.3 - 100.0 | 10.31 | 7.4 - 13.9 | 1.11 | 0.00 | 7.2 | 7.0 - 7.4 | 100.0 |  |
| >4 | 100.00 | 86.3 - 100.0 | 11.70 | 8.6 - 15.5 | 1.13 | 0.00 | 7.3 | 7.1 - 7.6 | 100.0 |  |
| >4.5 | 100.00 | 86.3 - 100.0 | 23.96 | 19.6 - 28.7 | 1.32 | 0.00 | 8.4 | 8.0 - 8.8 | 100.0 |  |
| >5 | 100.00 | 86.3 - 100.0 | 24.51 | 20.1 - 29.3 | 1.32 | 0.00 | 8.4 | 8.0 - 8.9 | 100.0 |  |
| >5.5 | 100.00 | 86.3 - 100.0 | 35.10 | 30.2 - 40.3 | 1.54 | 0.00 | 9.7 | 9.0 - 10.4 | 100.0 |  |
| >6 | 100.00 | 86.3 - 100.0 | 41.23 | 36.1 - 46.5 | 1.70 | 0.00 | 10.6 | 9.8 - 11.4 | 100.0 |  |
| >6.5 | 96.00 | 79.6 - 99.9 | 46.80 | 41.5 - 52.1 | 1.80 | 0.085 | 11.2 | 10.0 - 12.5 | 99.4 | 96.1 - 99.9 |
| >7 | 96.00 | 79.6 - 99.9 | 51.25 | 46.0 - 56.5 | 1.97 | 0.078 | 12.1 | 10.7 - 13.5 | 99.5 | 96.4 - 99.9 |
| >7.5 | 92.00 | 74.0 - 99.0 | 60.45 | 55.2 - 65.5 | 2.33 | 0.13 | 13.9 | 12.0 - 16.1 | 99.1 | 96.6 - 99.8 |
| >8 | 92.00 | 74.0 - 99.0 | 62.40 | 57.2 - 67.4 | 2.45 | 0.13 | 14.6 | 12.5 - 16.9 | 99.1 | 96.7 - 99.8 |
| **>8.5** | **84.00** | **63.9 - 95.5** | **71.59** | **66.6 - 76.2** | **2.96** | **0.22** | **17.1** | **14.0 - 20.7** | **98.5** | **96.3 - 99.4** |
| >9 | 72.00 | 50.6 - 87.9 | 80.22 | 75.7 - 84.2 | 3.64 | 0.35 | 20.2 | 15.5 - 25.9 | 97.6 | 95.6 - 98.7 |
| >9.5 | 72.00 | 50.6 - 87.9 | 82.17 | 77.8 - 86.0 | 4.04 | 0.34 | 22.0 | 16.8 - 28.1 | 97.7 | 95.7 - 98.8 |
| >10 | 60.00 | 38.7 - 78.9 | 89.97 | 86.4 - 92.9 | 5.98 | 0.44 | 29.4 | 21.1 - 39.4 | 97.0 | 95.2 - 98.1 |
| >10.5 | 36.00 | 18.0 - 57.5 | 93.04 | 89.9 - 95.4 | 5.17 | 0.69 | 26.5 | 15.9 - 40.7 | 95.4 | 94.0 - 96.6 |
| >11 | 24.00 | 9.4 - 45.1 | 98.61 | 96.8 - 99.5 | 17.23 | 0.77 | 54.5 | 28.2 - 78.5 | 94.9 | 93.7 - 95.9 |
| >11.5 | 12.00 | 2.5 - 31.2 | 98.89 | 97.2 - 99.7 | 10.77 | 0.89 | 42.9 | 15.1 - 76.0 | 94.2 | 93.3 - 94.9 |
| >12 | 0.00 | 0.0 - 13.7 | 99.72 | 98.5 - 100.0 | 0.00 | 1.00 | 0.0 |  | 93.5 | 93.4 - 93.5 |
| >12.5 | 0.00 | 0.0 - 13.7 | 100.00 | 99.0 - 100.0 |  | 1.00 |  |  | 93.5 | 93.5 - 93.5 |
